# Exosomal transfer of p-STAT3 promotes acquired 5-FU resistance in colorectal cancer cells

**DOI:** 10.1186/s13046-019-1314-9

**Published:** 2019-07-19

**Authors:** Qian Zhang, Rui-Xian Liu, Ka-Wo Chan, Jiancong Hu, Jingdan Zhang, Lili Wei, Huiliu Tan, Xiangling Yang, Huanliang Liu

**Affiliations:** 1 Guangdong Institute of Gastroenterology, The Sixth Affiliated Hospital, Sun Yat-sen University, Guangzhou, 510655 Guangdong China; 2 Guangdong Provincial Key Laboratory of Colorectal and Pelvic Floor Diseases, The Sixth Affiliated Hospital, Sun Yat-sen University, Guangzhou, 510655 Guangdong China; 3 Department of Clinical Laboratory, The Sixth Affiliated Hospital, Sun Yat-sen University, Guangzhou, 510655 Guangdong China; 4 Department of Colorectal Surgery, The Sixth Affiliated Hospital, Sun Yat-sen University, Guangzhou, 510655 Guangdong China

**Keywords:** P-STAT3, Exosomes, 5-FU resistance, Colorectal cancer

## Abstract

**Background:**

Acquired resistance remains a limitation of the clinical use of 5-fluorouracil (5-FU). Because exosomes, are important vesicles participating in intercellular communication, their contribution to the development of acquired 5-FU resistance needs to be elucidated. In this study, we aimed to examine the underlying mechanisms of exosomes from 5-FU resistant cells (RKO/R) in sustaining acquired 5-FU resistance in sensitive cells (RKO/P).

**Methods:**

Exosomes from a 5-FU-resistant cell line (RKO/R) and its parental cell line RKO/P were isolated and co-cultured with 5-FU-sensitive cells. Real-time cellular analysis (RTCA) and FACS analysis were used to examine cell viability and apoptosis. Exosomal protein profiling was performed using shotgun proteomics. Inhibitors and siRNAs were applied to study the involvement of selected proteins in 5-FU resistance. The effect of exosomal p-STAT3 (Tyr705) on the caspase cascade was examined by western blotting (WB) and high content analysis. Xenograft models were established to determine whether exosomal p-STAT3 can induce 5-FU resistance in vivo.

**Results:**

Our results indicated that exosomes from RKO/R cells significantly promoted cell survival during 5-FU treatment. Proteomics and WB analysis results indicated that GSTP1 and p-STAT3 (Tyr705) were enriched in exosomes from RKO/R cells. Inhibition of p-STAT3 re-sensitized RKO/P cells to 5-FU via caspase cascade. Furthermore, p-STAT3 packaged by exosomes from RKO/R cells increased resistance of tumor cells to 5-FU in vivo.

**Conclusions:**

Our results reveal a novel mechanism by which p-STAT3-containing exosomes contribute to acquired 5-FU resistance in CRC. This study suggests a new option for potentiating the 5-FU response and finding biomarkers for chemotherapy resistance.

**Electronic supplementary material:**

The online version of this article (10.1186/s13046-019-1314-9) contains supplementary material, which is available to authorized users.

## Background

Colorectal cancer (CRC) is one of the most common cancer in the world, ranking as the fourth diagnosed cancer [1]. Since 5-fluorouracil (5-FU) was discovered in 1957 [2], it has been used as first-line therapy in colorectal cancer [3]. Despite this, the efficacy of 5-FU-based chemotherapy is disappointing [4]. Most patients acquire resistance during treatment with a median survival of approximately 20 months [5, 6]. Thus, resistance to 5-FU-based chemotherapy has become the major hurdle to improve treatment efficacy.

The mechanisms of 5-FU resistance are complex. Some studies have demonstrated that cancer cells are resistant to chemotherapy intrinsically. Mutant p53 tumor suppressor gene is found in many human tumors and its activity is associated with 5-FU resistance [7]. Some members of Bcl-2 family, such as Bcl-xL and Bax, are related to 5-FU resistance in colorectal cancer [8]. Overexpression of Astrocyte elevated gene-1 (AEG-1) increases 5-FU resistance in human hepatocellular carcinoma (HCC) [9]. While other studies have indicated that drug resistance could be acquired. Tumor microenvironment includes many stromal cells and plays a critical role in chemoresistance. Cancer-associated fibroblasts (CAFs) are capable of decreasing drug uptake in tumors and causing resistance during chemotherapy [10]. Tumor associated macrophages (TAMs) could protect colorectal cancer cells from 5-FU-based chemotherapy via putrescine [11]. In triple-negative breast cancer, TGF-β increases the stem-like properties of cancer cells and causes drug-resistance [12].

Exosomes secreted by different kinds of cells are a kind of vesicles consisted of lipid bilayer membrane and play important roles in cell to cell communication [13]. These vesicles contain various proteins and nucleic acids including mRNAs and microRNAs [14, 15]. Recent studies have indicated that exosomes display multiple roles in tumor progression. Exosomes from mesenchymal stem cells could transfer angiogenesis-related microRNAs [16]. Exosomal microRNA-9 released by nasopharyngeal carcinoma cells inhibits angiogenesis by targeting MDK and is associated with good survival in patients [17]. MicroRNA-103 secreted by hepatoma cells is capable of promoting tumor cell metastasis [18]. Metastatic organotropism is associated with exosomes and the integrins of exosomes could be used to predict tumor metastasis [19]. Until now, most studies have focused on exosomal microRNAs transfer in various cancers. However, the mechanisms by which proteins in exosomes affect the phenotype of recipient cells have not been fully interpreted, especially in chemotherapy resistance.

Taken together, these findings promote us to investigate whether exosomes derived from 5-FU resistant cells could mediate 5-FU resistance in colorectal cancer cells. We verified the function of exosomes from 5-FU resistant cells RKO/R (Exo/R), analyzed the proteomics results of Exo/R and RNA-sequencing of 5-FU sensitive cells (RKO/P) and resistant cells (RKO/R). By doing this, we found that exosomal p-STAT3 enhanced 5-FU resistance in sensitive cells (RKO/P) via caspase cascade. Our results may open new avenues for discovering diagnostic markers and therapeutic targets for acquired 5-FU resistance.

## Methods

### Cell culture

Human colorectal cancer cell lines RKO and HCT116 were purchased from the Chinese Academy of Science (Shanghai, China). A 5-FU resistant cell line (RKO/R) was established from RKO parental cell line (RKO/P) as previously described [20]. RKO/P and RKO/R cells were cultured in DMEM (Gibco, USA), and HCT116 cells were cultured in RPMI-1640 medium (Gibco, USA). RKO/R cells were cultured without 5-FU for this experiment. All medium contained 10% FBS (Gibco USA) and a penicillin-streptomycin solution (Gibco, USA). Cells were incubated in a humidified incubator with 5% CO_2_ at 37 °C. For cell apoptosis assays, cells were seeded in 48-well plates. After 24 h, 200 μl of culture medium containing 5-FU, DMSO, inhibitors (Table 1) and exosomes was added to each well. Cells were then harvested for FACS analysis at different time points.

**Table 1:** Reagents list

Reagents	Product number	Company
5-fluorouracil	F8423-5G	Sigma-Aldrich (USA)
DMSO	D2650	Sigma-Aldrich (USA)
ezatiostat	A8225	APExBIO Technology (USA)
stattic	A2224	APExBIO Technology (USA)

### Exosome isolation and normalization

Exosomes were extracted from the same volume of culture medium without FBS. The supernatant was centrifuged at 300 g for 10 min at 4 °C and then at 1,000 g for 10 min at 4 °C to remove apoptotic bodies, followed by 10,000 g for 30 min at 4 °C. Finally, the supernatant from the former step was centrifuged by 100,000 g for 70 min at 4 °C using an ultracentrifuge (Beckman Coulter, USA). After centrifugation, the exosomes were resuspended in complete culture medium or phosphate-buffered saline (PBS). Exo/P or Exo/R came from 10^6^ RKO/P or RKO/R cells was added to each well of 48-well plates.

### Flow cytometry

Apoptosis assays were performed after 72 h, and the cells were harvested according to the manufacturer’s instructions using an Annexin V-APC/7-AAD staining kit (MultiSciences, China). The apoptotic rate of the cells was determined by flow cytometry (BD FACSCanto, USA). All data were exported as FCS 3.0 documents and analyzed with FlowJo software 10.0.7. Cell cycle assays were performed after 24 h, and cells were harvested according to the manufacturer’s instructions for the cell cycle staining buffer (MultiSciences, China).

### BrdU proliferation assay

Cells were plated in 96-well plates and incubated for 24 h. Then, BrdU, 5-FU and exosomes were added to each well and incubated for 24 h. Cell proliferation was measured using a BrdU cell proliferation ELISA kit (Abcam, UK). The absorbance of the samples was measured according to the manufacturer’s instructions.

### Transmission electron microscopy (TEM)

Exosomes were resuspended in PBS and placed onto copper grids at room temperature. Extra solution was removed with filter paper, and phosphotungstic acid solution was added. Then, filter paper was used again to remove the excess solution. The copper grids were dried at room temperature for 2 min, and the exosomes were observed with transmission electron microscopy (JEM-1200EX, Japan).

### High sensitivity flow cytometry (HSFCM)

To determine size distribution, exosomes were resuspended with PBS after ultracentrifugation and detected with a Flow NanoAnalyzer (NanoFCM, China).

### Silver staining and shotgun proteomics

Exosomes were lysed with SDT buffer (4% SDS, 100 mM DTT, 150 mM Tris-HCl) and boiled for 5 min. The same volume of each sample was loaded. Exo/P and Exo/R proteins were visualized using a silver staining kit (Beyotime, China). Exo/R were lysed with SDT buffer and boiled for 15 min for mass spectrometry. Then, detergent, DTT and other low-molecular-weight components were removed, and the protein suspensions were digested at 37 °C overnight. The resulting peptides were desalted on C18 cartridges (Sigma, USA). Mass spectrometry was performed on a Q Exactive mass spectrometer (Thermo Scientific, USA). Finally, the data were analyzed with MaxQuant software version 1.5.3.17 (Max Planck Institute of Biochemistry, Germany) and searched against the UniProt database.

### RNA-sequencing

Total RNA was extracted from RKO/P and RKO/R. RNA molecules were purified using oligo (dT) attached magnetic beads. Then, mRNA was fragmented into small pieces using fragmentation reagent. For cDNA synthesis, first-strand cDNA was generated using random N6 primers, followed by second strand synthesis. PCR was used to amplify the cDNA fragments with adaptors from the previous step. The double stranded PCR products were heat separated, and the single strand circle RNA was formatted as the final library. RNA-seq libraries were sequenced using a BGISEQ-500 sequencer. Raw reads that contained the sequences of adaptor and low-quality reads were filtered before downstream analysis. Clean reads were mapped to reference genes using Bowtie2. Gene expression levels were quantified by the RSEM software package, and NOISeq software was used to screen differentially expressed genes.

### WB analysis

Cells and exosomes extracts were lysed with RIPA buffer (Beyotime, China) containing protease inhibitor (KeyGEN Biotech, China). Equal amounts of protein samples were loaded and run on SDS-PAGE gels equally and transferred to Hybridization Nitrocellulose Filter (Merck Millipore, Germany). Then, the membranes were blocked with 5% skim milk (BD Biosciences, USA) dissolved in 0.1% Tris-buffered saline with Tween-20 (0.1% TBST) at room temperature for 1 h. Next, the membranes were incubated with primary antibodies (Table 2) diluted in primary antibody solution (Toyobo, Japan) overnight at 4 °C. On the following day, 0.1% TBST was used to wash the membranes. Then, the membranes were incubated with secondary antibodies diluted at 1:5000 in 0.1% TBST at room temperature for 2 h. HRP-conjugated goat anti-rabbit IgG (H + L) (Thermo Scientific, USA) and goat anti-mouse IgG (H + L) (Thermo Scientific, USA) were used for the secondary antibodies. Signals were developed with ECL (Santa Cruz, USA) and protein bands were visualized on X-ray film.

**Table 2:** Primary antibody list

Name	Product number	Company	Dilution (WB)	Dilution (IF)
ALIX	GTX42812	Genetex (USA)	1:200	
Caspase-9	9502	Cell Signaling Technology (USA)	1:1000	
Caspase-3	9662	Cell Signaling Technology (USA)	1:1000	
CD63	ab59479	Abcam (UK)	1:200	
CD9	ab92726	Abcam (UK)	1:200	
GAPDH	10494–1-AP	Proteintech (USA)	1:1000	
GSTP1	GTX112695	Genetex (USA)	1:1000	1:200
LaminB1	12987–1-AP	Proteintech (USA)	1:1000	
p-STAT3 (Tyr705)	9145	Cell Signaling Technology (USA)	1:200	1:200
STAT3	9139	Cell Signaling Technology (USA)	1:1000	
TBP	44059	Cell Signaling Technology (USA)	1:1000	
TSG101	ab125011	Abcam (UK)	1:200	
β-actin	60008–1-Ig	Proteintech (USA)	1:1000	

### Confocal microscopy analysis

RKO/P cells were cultured in confocal dishes (NEST, China). Exo/P and Exo/R were labeled with the fluorescent dye PKH-26 (Sigma, USA) following the manufacturer’s instructions. Then, exosomes were ultracentrifuged again. RKO/P cells were incubated with the PKH-26 labeled Exo/P and Exo/R. Cells were fixed with 4% paraformaldehyde for 20 min, and washed with PBS containing 0.1% Tween (0.1% PBST) for confocal microscopy. Cell membranes were permeabilized with 0.2% Triton X-100 or methanol for 10 min and washed with 0.1% PBST. For immunofluorescence, cells were incubated with QuickBlock™ Blocking Buffer for Immunol Staining (Beyotime, China) for 1 h at room temperature and were then incubated with primary antibodies (Table 2) diluted in QuickBlock™ Primary Antibody Dilution Buffer for Immunol Staining (Beyotime, China) at 4 °C in a humidified chamber overnight. Subsequently, the dishes were washed and incubated with anti-rabbit or anti-mouse Alexa Fluor® Plus 488 secondary Antibody (1:500, Thermo Scientific, USA) at room temperature for 2 h. Cell nuclei were labeled with DAPI (Thermo Scientific, USA) for 2 min. Finally, the dishes were observed under a laser scanning confocal microscope (Leica TCS-SP8, Germany).

### Real-time cellular analysis (RTCA)

RTCA (ACEA Biosciences, USA) was used to monitor cell viability, and was performed according to the manufacturer’s instructions. Cell index is used to monitor cell status including cell numbers and cell attachment. When cells adhere to the surface of E-plate and influence the electrical impedance across the array, the xCELLigence software records electrical values and converts it into cell index [21]. First, 50 μl of culture medium was added to measure the background value. Then, the cells were mixed with 50 μl of culture medium and seeded into E-plates. Culture medium containing exosomes, 5-FU, DMSO and inhibitors was added when the cell index reached 1.0. The data were documented and exported with ACEA Biosciences RTCA software 2.0 and analyzed by Microsoft Excel and GraphPad Prism 7.0.

### siRNA transfection

Chemically synthesized GSTP1 and STAT3 siRNAs (Table 3) and control siRNAs were purchased from RiboBio (China). The sequences of the siRNAs are shown in Table 3. The siRNAs were transiently transfected into RKO/P and RKO/R cells using Lipofectamine RNAiMAX (Invitrogen, USA) according to the manufacturer’s instructions. After 48 h of transfection, the cells were analyzed by WB, and the conditional medium was collected.

**Table 3:** siRNA sequences

Gene		Sequence
GSTP1	si-1	CCTACACCGTGGTCTATTT
	si-2	TACATCTCCCTCATCTACA
STAT3	si-1	CCGTGGAACCATACACAAA
	si-2	CATCTGCCTAGATCGGCTA

### Caspase-3 activity assay

Cells were seeded in 96-well plates, and culture medium containing exosomes, 5-FU, DMSO and inhibitors were added after 24 h. Caspase-3 activity was determined per well according to the manufacturer’s instructions for the GreenNuc™ Caspase-3 activity assay kit (Beyotime, China). Fluorescence images were obtained using an Operetta CLS™ high-content cell imaging analysis system (PerkinElmer, USA), and a 20× objective lens was used in our experiment. The percentage of caspase-3 was calculated by the software Harmony 4.5 and GraphPad Prism 7.0.

### Animal experiments

Animal experiments were approved by the Committee on the Ethics of Animal Experiments of The Sixth Affiliated Hospital, Sun Yat-sen University. Male BALB/c nude mice (4–5 weeks) were purchased from Charles river (China). RKO/P cells were injected to the right flank of each mouse subcutaneously (3 × 10^6^ cells in 200 μl PBS per mouse). All mice were divided into four groups (Exo/P + DMSO, Exo/P + stattic, Exo/R + DMSO, Exo/R + stattic) when tumors reached volume of 50–100 mm^3^. 5-FU (50 mg/kg) and stattic (25 mg/kg) were administered intraperitoneally (i.p.) every 2 days. Exosomes (10 μg) were injected into the xenograft tumors every 4 days. Tumor volumes and body weights were measured every 2 days. The mice were sacrificed after 17 days of treatment, and the size and weight of tumors from each group were measured.

### Statistical analysis

Statistical analysis was performed with the GraphPad Prism 7 software. All data were presented for two replicates or three times. Statistical significance was determined by Student’s t-test for two groups. We also used One-way ANOVA multiple comparison analysis with Tukey’s posttest. Two-way ANOVA with Fisher’s LSD test was used to compare more groups. *P* < 0.05 was regarded as statistically significant.

## Results

### Exosomes from RKO/R cells attenuated the chemosensitivity of 5-FU-sensitive cells

**Fig. 1:**
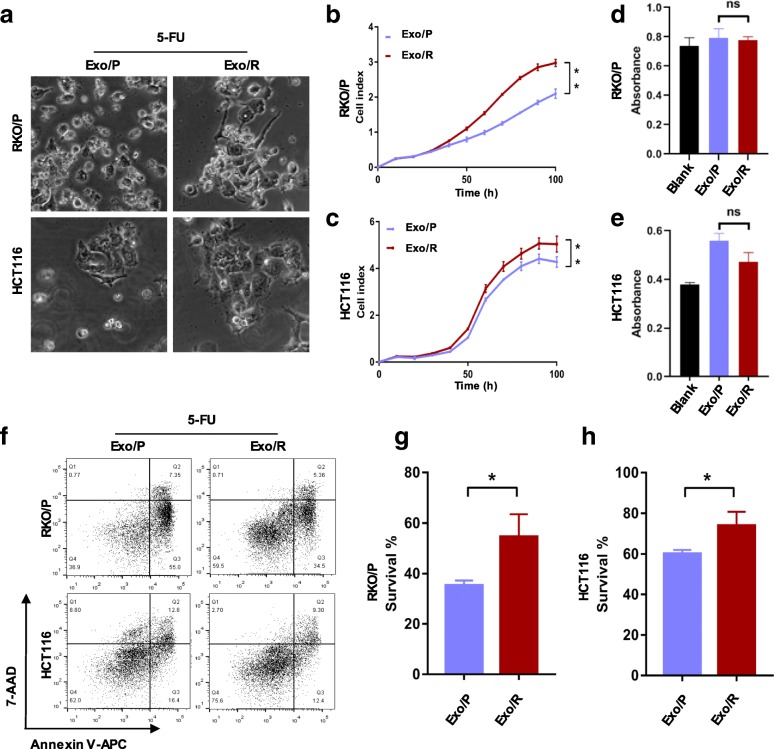
Exo/R increased 5-FU resistance of RKO/P and HCT116 cells. **a**. Pictures of RKO/P and HCT116 cells treated with 30 μM and 10 μM 5-FU and exosomes after 72 h. **b**, **c**. RTCA of RKO/P (**b**) and HCT116 (**c**) cells treated with exosomes and exposed to 30 μM and 10 μM 5-FU. **d**, **e**. The absorbance showed the percentage of S-phase in RKO/P (**d**) and HCT116 (**e**) cells treated with 30 μM and 10 μM 5-FU and exosomes for 24 h. f. Representative apoptotic RKO/P and HCT116 cells treated with 30 μM and 10 μM 5-FU and exosomes for 72 h. Apoptotic cells were stained with Annexin V-APC and 7-AAD. **g**, **h**. Quantitative analysis of survival rate of RKO/P and HCT116 by FACS. *P < 0.05, **P < 0.01

Studying the mechanisms of chemotherapy resistance remains a challenge due to tumor heterogeneity and the complex tumor microenvironment. We interpreted it in colorectal cancer cells utilizing 5-FU resistant cells (RKO/R) established from 5-FU sensitive cells (RKO/P). To determine the capability of exosomes isolated from RKO/R cells to transmit acquired 5-FU resistance to recipient cells, we first cultured recipient cells (RKO/P and HCT116) in medium containing 5-FU plus Exo/P or Exo/R. After 72 h, we observed the cells under a microscope and found that there were more survival cells attached the plate upon treatment with Exo/R and 5-FU (Fig. 1a). To analyze this change, we used RTCA to monitor cell viability continuously. The results showed that the cell index of the recipient cells incubated with Exo/R was significantly higher than that of the recipient cells incubated with Exo/P (Fig. 1b, c). To determine whether the cell index was altered by reduced cell death or increased cell proliferation, we next measured cell proliferation and cell apoptosis in the recipient cells. Notably, although the cell cycle was significantly arrested by 5-FU, Exo/R did not alter the cell cycle distribution significantly (see Additional file 1: Figure S1). To further interpret cell proliferation in recipient cells, BrdU was used to measure cell cycle blockade at S-phase. Exo/R did not alter S-phase arrest induced by 5-FU in RKO/P and HCT116 cells importantly (Fig. 1d, e). However, the cell apoptosis assay results indicated that Exo/R significantly promoted cell survival compared to Exo/P (Fig. 1f, g, h). Thus, we proved that exosomes from resistant cells were responsible for acquired 5-FU resistance by decreasing apoptosis in recipient cells.

### The characteristics of Exo/P and Exo/R

**Fig. 2:**
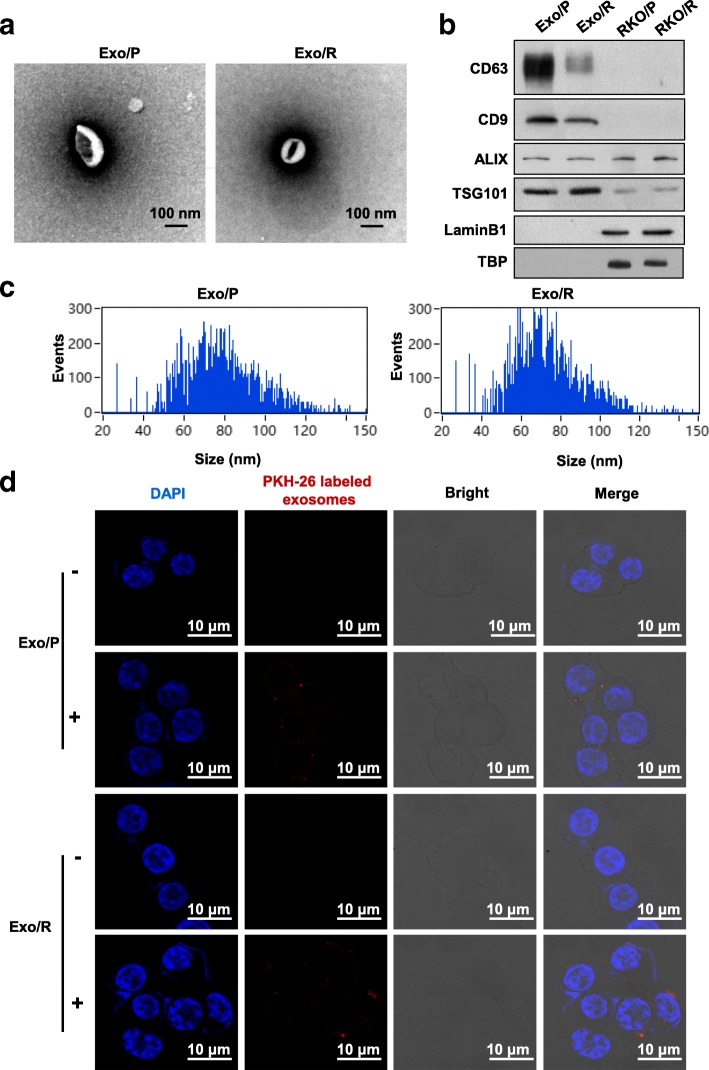
The identification of exosomes and uptake of exosomes by RKO/P cells. **a**. Representative images of exosomes derived from RKO/P and RKO/R taken by TEM (scale bar, 100 nm). **b**. Exosomal markers (CD63, CD9, ALIX, TSG101) and non-exosomal makers (LaminB1, TBP) were analyzed by WB in Exo/P, Exo/R, RKO/P and RKO/R. **c**. Histogram indicated diameter of exosomes purified from RKO/P and RKO/R analyzed by HSFCM. **d**. Confocal images of cells incubated with (+) or without (−) PKH-26 labelled Exo/P and Exo/R for 6 h. (scale bar, 10 μm)

As Exo/P and Exo/R showed different abilities to induce 5-FU resistance, we hypothesized that their characteristics were different. Exosomes were isolated from an equal volume of conditional medium collected from RKO/P and RKO/R cells and characterized by TEM, WB and HSFCM. As expected, typical cup-shaped morphology and similar size distribution were confirmed in both Exo/P and Exo/R (Fig. 2a, c). In addition, the exosomal markers CD63, CD9 and ALIX were enriched in Exo/P and Exo/R while TSG101 was detected in both cells and exosomes. Moreover, the absence of the nuclear markers LaminB1 and TBP in Exo/P and Exo/R indicated the purity of the exosomes in our experiment (Fig. 2b). We next examined whether these exosomes were taken up by recipient cells. Purified Exo/P and Exo/R were labeled with PKH-26 (red fluorescence) and incubated with recipient cells. After 6 h, the localization of exosomes in cells was observed by confocal microscopy (Fig. 2d). Collectively, these results suggested that Exo/R mediated 5-FU resistance may be associated with exosome internalization.

### Identification of chemoresistant proteins in exosomes

**Fig. 3:**
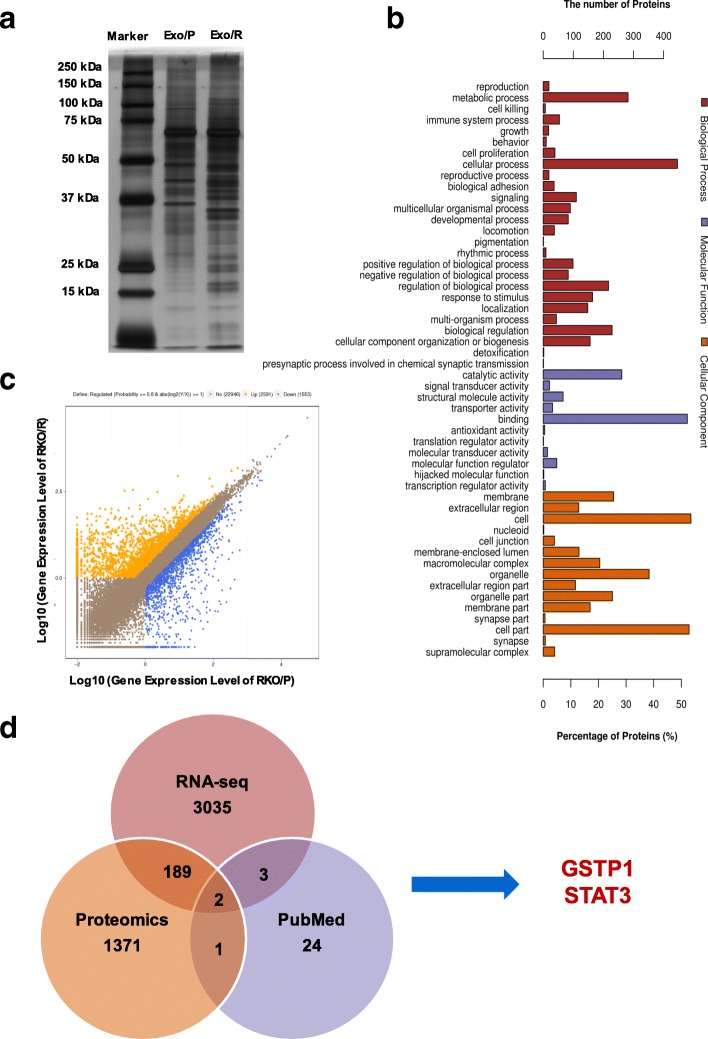
Identification of candidate proteins associated with 5-FU resistance. **a**. SDS-PAGE and silver-staining showed the different proteins in Exo/P and Exo/R. **b**. GO analysis of proteins of Exo/R. Proteins were categorized with the Biological Processes, Molecular functions and Cellular Components. **c**. Scatterplot showed the alterations in RKO/R and RKO/P. Yellow and blue spots indicated the up-regulated and down-regulated genes. **d**. Venn diagram indicated the shared and unique genes names among RNA-seq, Proteomics (proteins were converted to their corresponding gene names on Uniprot) and the search results on PubMed

We hypothesized that the proteins encapsulated in exosomes are involved in 5-FU resistance. To validate this hypothesis, we utilized SDS-PAGE and silver staining to fractionate and detect the proteins in exosomes. Notably, the protein profile displayed abundant distinct protein bands for Exo/P and Exo/R (Fig. 3a). To further determine 5-FU resistance related exosomal proteins, shotgun proteomics analysis was performed on Exo/R. The raw data were processed using the human Uniprot database and 964 proteins were identified in Exo/R. Next, bioinformatic tools were used to characterize the function of proteins in Exo/R. A total of 918 proteins were analyzed for gene ontology (GO) annotation and predicted to be involved in the biological processes, molecular functions and cellular components (Fig. 3b). To identify the key proteins involved in exosome-mediated 5-FU resistance, RNA-sequencing was performed on RNA samples extracted from RKO/P and RKO/R and differentially expressed RNAs were plotted (Fig. 3c). After combining the results of 964 identified proteins and 3229 up-regulated RNAs (probability> 0.69), 192 gene symbols were screened (Additional file 2: Table S1). Among these results, we searched for reported chemoresistance associated proteins and selected GSTP1 and STAT3 for further study (Fig. 3d).

### GSTP1 and p-STAT3 are nuclear proteins enriched in Exo/R

**Fig. 4:**
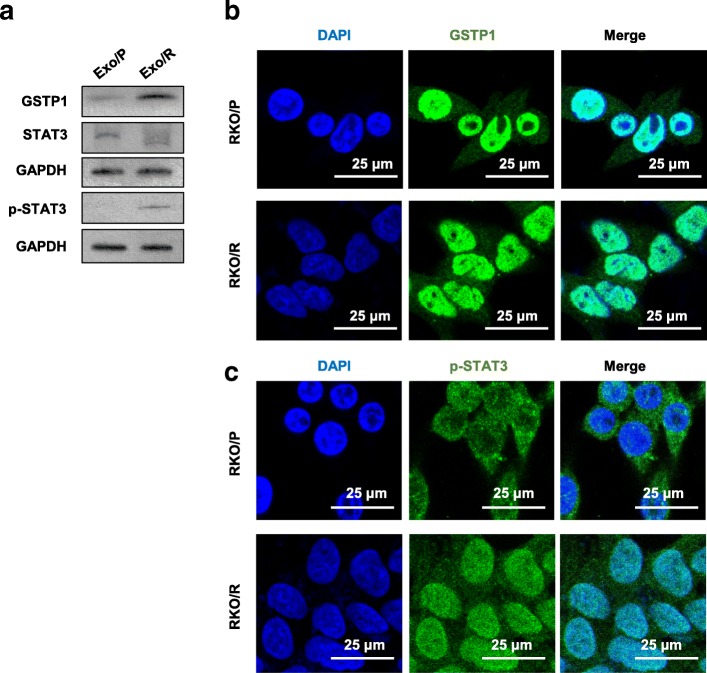
The detection of GSTP1 and p-STAT3 in exosomes and cells. **a**. The detection of GSTP1, STAT3 and p-STAT3 in Exo/P and Exo/R. **b**, **c**. Immunofluorescence of GSTP1 and p-STAT3 in RKO/P and RKO/R. (scale bar, 25 μm)

To validate whether GSTP1 and STAT3 were enriched in Exo/R, we lysed Exo/P and Exo/R. Compared with that in Exo/P, increased expression of GSTP1 but not STAT3 was detected in Exo/R. Notably, we also detected p-STAT3 in Exo/R, which could not be detected in Exo/P (Fig. 4a). p-STAT3 and GSTP1 are known to be located mainly in the cell nucleus. We next explored the distribution of GSTP1 and p-STAT3 in RKO/P and RKO/R cells. Immunofluorescence showed that GSTP1 was expressed mainly in cell nucleus and partially expressed in the cytoplasm of RKO/P and RKO/R cells (Fig. 4b). p-STAT3 was expressed in both the nucleus and cytoplasm of RKO/R cells but not in the nucleus of RKO/P cells (Fig. 4c). These observations suggested that GSTP1 and p-STAT3 are enriched in Exo/R and may be responsible for exosome-mediated 5-FU resistance.

### P-STAT3 is required for exosome-mediated 5-FU resistance

**Fig. 5:**
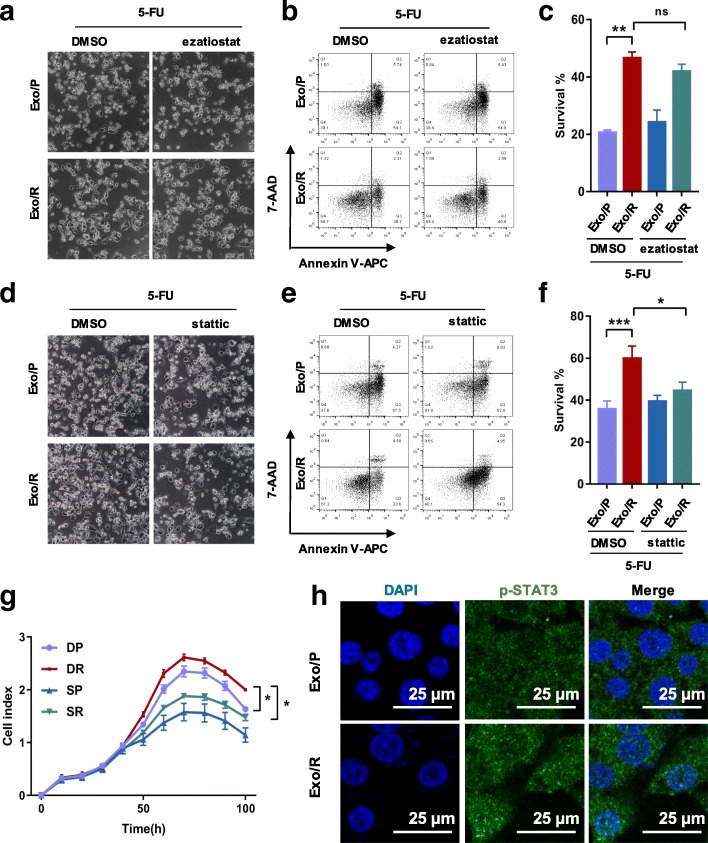
The verification of p-STAT3 transferred by Exo/R and its anti-apoptotic function in RKO/P cells. **a**, **b**, **c**. Representative images of RKO/P treated with 30 μM 5-FU, 10 μM ezatiostat and exosomes for 72 h and statistical analysis of the survival rate. **d**, **e**, **f**. Representative images of RKO/P cells treated with 30 μM 5-FU, 7.5 μM stattic and exosomes for 72 h and statistical analysis of the survival rate. **g**. RTCA analysis of cell index of RKO/P cells treated with 30 μM 5-FU, Exo/P and Exo/R, DMSO or 5 μM stattic. DP: DMSO + Exo/P, DR: DMSO + Exo/R, SP: stattic + Exo/P, SR: stattic + Exo/R. **h**. Confocal images of p-STAT3 in RKO/P cells co-cultured with Exo/P or Exo/R in the absence of 5-FU. (scale bar, 25 μm) *P < 0.05, **P < 0.01, ***P < 0.001

To further investigate whether exosomal GSTP1 or p-STAT3 is involved in acquired 5-FU resistance, we used protein inhibitors in combination with exosomes to treat recipient cells. We first evaluated the effect of exosomal GSTP1 in mediating 5-FU resistance. The results showed that neither the morphology nor the survival rate of cells treated with Exo/R and a GSTP1 inhibitor (ezatiostat) changed significantly compared to those of cells treated with Exo/R and DMSO (Fig. 5a, b, c), excluding the possibility that 5-FU resistance is mediated by GSTP1 in exosomes. Moreover, both apoptotic morphology and reduced cell survival demonstrated that a STAT3 inhibitor (stattic) abolished 5-FU resistance induced by Exo/R (Fig. 5d, e, f). The effect of exosomal GSTP1 and p-STAT3 in mediating acquired 5-FU resistance was also verified by siRNA. Down-regulating p-STAT3 in RKO/R cells markedly abolished the anti-apoptotic function of Exo/R (Additional file 1: Figure S2). A significant decrease in the cell index also supported the effect of exosomal p-STAT3 on 5-FU resistance (Fig. 5g). Furthermore, immunofluorescence was carried out to stain p-STAT3 in recipient cells. We co-cultured Exo/P or Exo/R with recipient cells for 72 h and found that more p-STAT3 was expressed in the cell nucleus (Fig. 5h). Taken together, these results showed that p-STAT3 transfer via exosomes is responsible for acquired 5-FU resistance, and that p-STAT3 inhibition can restore the sensitivity of recipient cells to 5-FU.

### The caspase pathway is responsible for p-STAT3-mediated 5-FU resistance

**Fig. 6:**
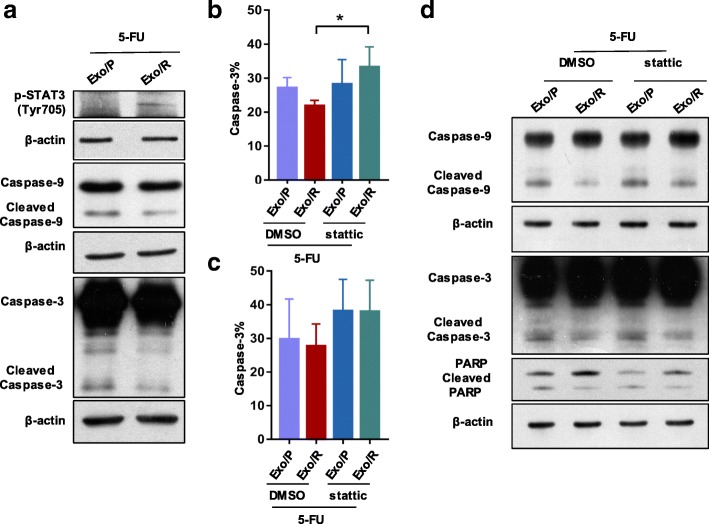
Caspase pathway was activated by the inhibition of p-STAT3 in Exo/R. **a**. RKO/P cells were exposed to 30 μM 5-FU and exosomes for 72 h, and then WB was used to detect the expression of p-STAT3, cleaved Caspase-9 and Caspase-3. b, c. High content analysis of active caspase-3 in RKO/P cells. Caspase-3 activity was calculated at 36 h (**b**) and 72 h (**c**) after cells exposed to 30 μM 5-FU, exosomes and 5 μM stattic. d. RKO/P cells were cultured with 30 μM 5-FU and exosomes in the presence of DMSO or 5 μM stattic for 56 h. WB was used to detect cleaved caspase-9, caspase-3 and PARP in RKO/P cells. * *P* < 0.05

To clarify the molecular mechanisms by which exosomes contribute to 5-FU resistance in CRC, we investigated the caspase cascade in recipient cells. We first confirmed that p-STAT3 was increased in cells treated with Exo/R by WB. In addition, less cleaved caspase-9 and caspase-3 expression demonstrated that Exo/R decreased cell apoptosis when exposed to 5-FU (Fig. 6a). To demonstrate that stattic overcomes Exo/R-mediated 5-FU resistance, we assessed caspase-3 activity in RKO/P cells. The results showed that activated caspase-3 increased after exposure to stattic for 36 h and 72 h compared to that after DMSO exposure (Fig. 6b, c). Consistent with this finding, the apoptosis-related proteins caspase-9, caspase-3, and PARP were also determined in recipient cells using WB. Cleaved caspase-9 increased obviously after treatment with stattic. Cleaved caspase-3 and PARP were also detected by WB (Fig. 6d). These data highlighted the role of the caspase cascade in 5-FU resistance mediated via exosomal p-STAT3.

### Exosomes from RKO/R cells induce 5-FU resistance in RKO/P cells in vivo

**Fig. 7:**
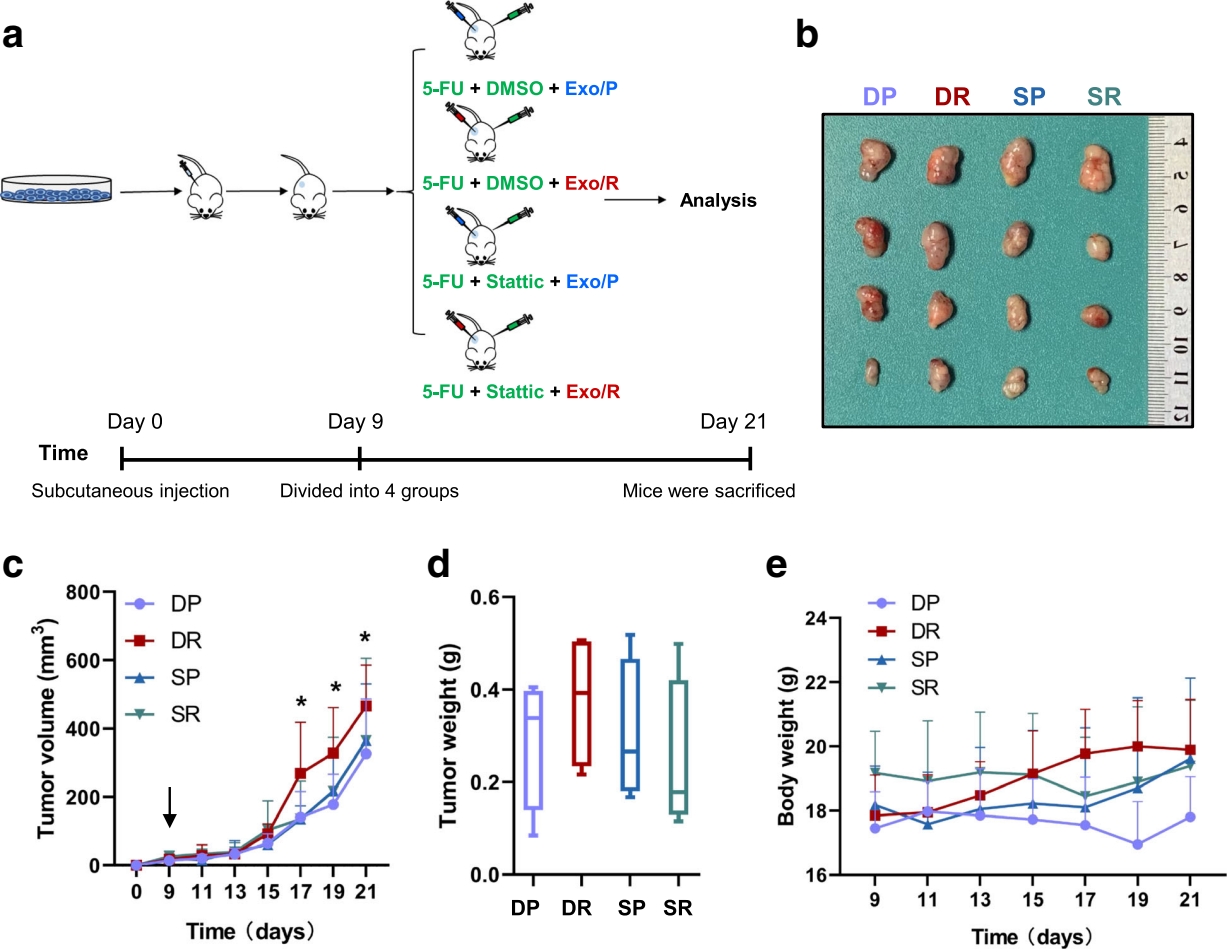
Exosomal p-STAT3 derived from RKO/R cells induce 5-FU resistance in RKO/P cells in vivo. **a**. Flowchart of mice experiment. **b**. Tumors picture from mice treated with DP (DMSO + Exo/P), DR (DMSO + Exo/R), SP (stattic + Exo/P), SR (stattic + Exo/R). **c**. Tumor growth curves of mice treated with DP (DMSO + Exo/P), DR (DMSO + Exo/R), SP (stattic + Exo/P), SR (stattic + Exo/R). Treatment started on Day 9 and tumor volumes was measured every 2 days. **d**. The tumor weights from mice treated with DP (DMSO + Exo/P), DR (DMSO + Exo/R), SP (stattic + Exo/P), SR (stattic + Exo/R). **e**. The curves of body weights of mice treated with DP (DMSO + Exo/P), DR (DMSO + Exo/R), SP (stattic + Exo/P), SR (stattic + Exo/R). (*n* = 4, *P < 0.05)

To further determine whether exosomal p-STAT3 can induce 5-FU resistance in colorectal cancer in vivo, we established a subcutaneous xenograft model in BALB/c nude mice with RKO/P cells. 5-FU was injected into each group together with exosomes (Exo/P or Exo/R) and stattic or DMSO (Fig. 7a). The sizes of subcutaneous tumors treated with Exo/R were larger than those treated with Exo/P under 5-FU therapy, indicating that Exo/R inhibited the effect of 5-FU and promoted tumor growth. In addition, the volumes of tumors treated with Exo/R plus stattic were not different from those of tumors treated with Exo/P, but were smaller than those treated with Exo/R plus DMSO (Fig. 7b, c). Furthermore, the mean tumor weights of mice treated with Exo/R were heavier than those of mice in other groups (Fig. 7d). The body weights of mice treated with Exo/R were a little heavier than other groups (Fig. 7e). Taken together, these results indicated the function of p-STAT3 encapsulated by Exo/R in regulating 5-FU resistance in colorectal cancer cells in vivo.

## Discussion

5-FU-based chemotherapy significantly prolonged the life expectancy of patients, however, resistance to 5-FU is a major limitation to treatment success [22]. Due to complicated and variable biological processes, the mechanisms of chemoresistance are still elusive [23]. It is known that the functions of exosomes are not restricted to maintaining normal biological processes but also encompass drug resistance. Previous studies have shown that exosomes secreted by bone marrow stromal cells (BMSCs), CAFs and tumor cells promote chemotherapy resistance in human tumors [24–26]. In this study, we isolated exosomes from 5-FU-resistant cells, identified exosomal p-STAT3 with shotgun proteomics analysis and verified its function in 5-FU resistance. These findings demonstrated an unconventional mechanism of acquired drug resistance in colorectal cancer.

Exosomes play multiple roles in intercellular communication by transmitting RNAs and proteins [27, 28]. In previous studies, exosomes were described as important vesicles disseminating drug resistance. MicroRNAs in exosomes, which could change various pathways related to chemotherapy resistance have been reported in different cancers. For instance, cisplatin resistance in lung cancer is associated with exosomal miR-100-5p [29], and the PI3K/Akt pathway in hepatocellular carcinoma (HCC) is activated by miR-32-5p delivered by exosomes from resistant cells [30]. Efflux of the tumor-suppressors miR-145 and miR-34a via microvesicles is responsible for 5-FU resistance in colon cancer cells [31]. Notably, the transmission of proteins by exosomes is significant in regulating chemotherapy resistance. For instance, some researchers have shown that TrpC-5-containing extracellular vesicles in breast cancer and P-glycoprotein (P-gp)-containing microvesicles in ovarian cancer are responsible for chemotherapeutic resistance [32, 33]. GSTP1, which is associated with detoxification and glutathione conjugation, has been reported in adriamycin-resistant breast cancer cells [34, 35]. However, in the above studies, the functional proteins were selected by subjective conjecture instead of screening objectively. Thus, only some well-known proteins were identified and novel and pivotal components in the exosomes were not explored. In contrast, we performed proteome profiling analysis and subsequent validation studies. Our results demonstrated for the first time that p-STAT3 accumulation in exosomes is relevant to 5-FU resistance. More importantly, the results suggested that more large-scale mass spectrum-based analyses should be performed to screen potential chemoresistant proteins in exosomes.

Previous study has found that IL-6 could induce STAT3 phosphorylation and subsequently regulate transcription [36]. STAT3 is activated in many cancers and associates with patient’s survival [37]. In addition, increased p-STAT3 levels have been reported in CRC and correlated with chemoradiotherapy [38]. Recent studies revealed that the inhibition of STAT3 sensitized colorectal cancer cells to 5-FU treatment through down-regulating cyclinD1 [39]. Nevertheless, few studies have focused on exosomal p-STAT3 in colorectal cancer and the selectivity of proteins transported by exosomes has not been fully understood. A previous study indicated that cells treated with 5-FU were larger than control cells, and their nuclear staining was pale [40]. Our results showed that the nuclei of RKO/R cells appeared irregular shape and had pale DAPI staining. In brief, our findings not only illustrated the enrichment of p-STAT3 in exosomes but also implied the possible mechanisms that regulate this enrichment in exosomes.

Stattic, which selectively inhibits the activation, dimerization and nuclear translocation of STAT3, was used to prevent p-STAT3 from translocating to the nucleus [41]. In our study, p-STAT3 was enriched in Exo/R and participated in exosome-mediated 5-FU resistance. These results suggested that developing inhibitors that selectively impair the function of p-STAT3 would be an effective way to reduce exosome-mediated chemotherapy resistance. Additionally, more evidence suggests that exosomes could be used as promising biomarkers to diagnose cancer [42]. However, their application in diagnosing chemotherapy resistance is still lacking. Thus, the application of p-STAT3 as a potential biomarker would help to monitor the 5-FU resistance of patients during treatment. More investigations are needed to validate the clinical use of p-STAT3-containing exosomes as a therapeutic target and biomarker in colorectal cancer.

## Conclusions

**Fig. 8:**
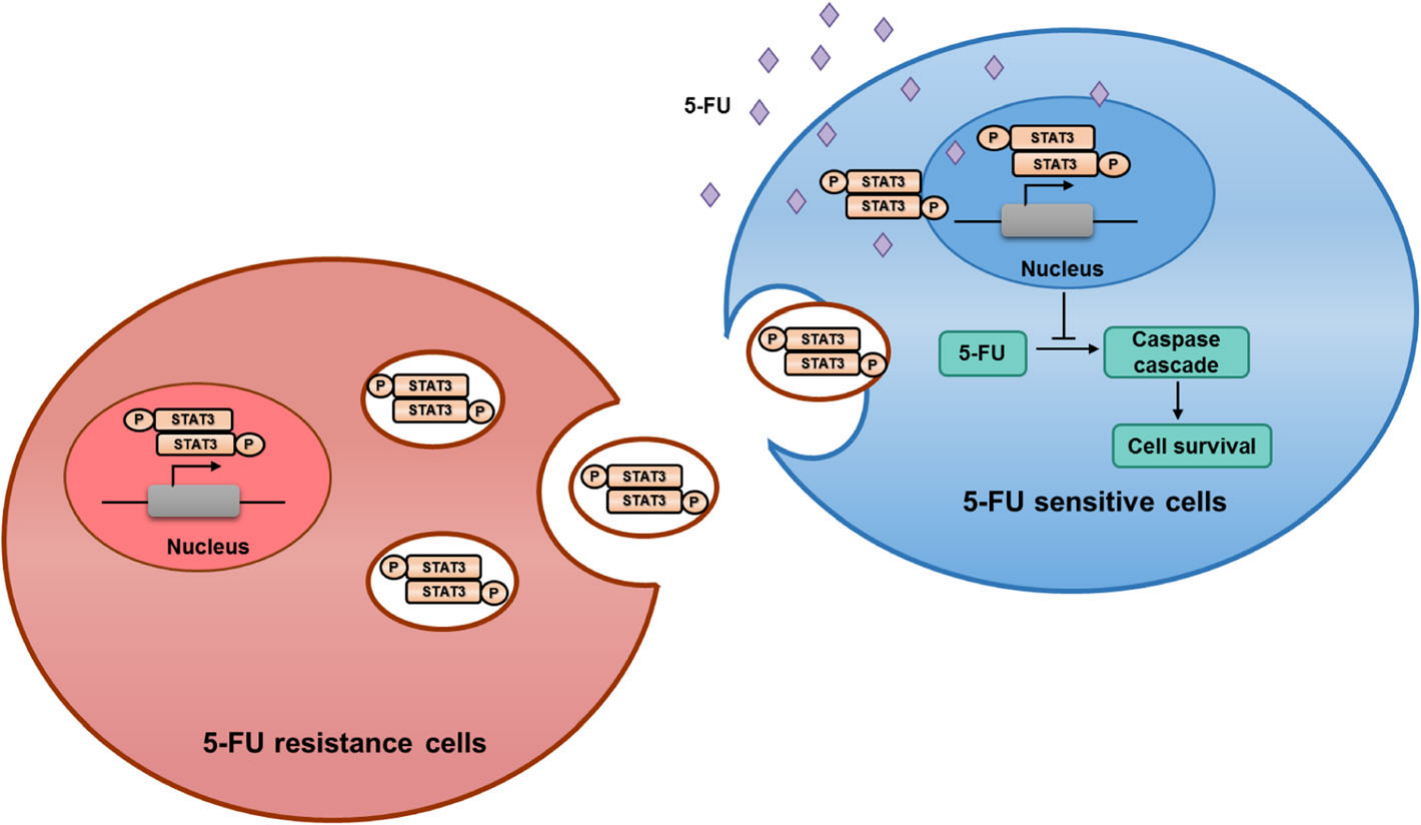
Schematic representation of exosomal p-STAT3 in regulating 5-FU resistance. A schematic diagram indicated how exosomes from RKO/R cells mediated 5-FU resistance in sensitive cells

In summary, our findings prove that p-STAT3 transferred by exosomes from 5-FU-resistant cells could induce chemotherapy resistance in recipient cells by reducing caspase cascade activation (Fig. 8).

## Additional files

Additional file 1: **Figure S1.** The cell cycle distribution of RKO/P and HCT116 cells. a. Representative cell cycle changes of HCT116 and RKO/P cells exposed to 10 μM or 30 μM 5-FU and Exo/P or Exo/R after 24 hours. The DNA content was stained with PI. exposed to 10 μM or 30 μM 5-FU and Exo/P or Exo/R after 24 hours. The DNA content was stained with PI. **Figure S2.** p-STAT3 transferred by Exo/R p-STAT3 transferred by Exo/R mediated acquired 5-FU resistance in RKO/P cells. **a**. The WB result of GSTP1 and STAT3 in RKO/P and RKO/R cells transfected with si-NC, si-GSTP1 (si-1, si-2) and si-STAT3 (si-1, si-2). **b**. Statistical analysis of the survival rate of RKO/P cells treated with 30 μM 5-FU and exosomes from RKO/P or RKO/R cells after down-regulation of GSTP1. **c**. Statistical analysis of the survival rate of RKO/P cells treated with 30 μM 5-FU and exosomes from RKO/P or RKO/R cells after down-regulation of p-STAT3. *P < 0.05, **P < 0.01. (PDF 308 kb)

Additional file 2: **Table S1.** Overlapped parts in Venn diagram among RNA-seq, Proteomics and PubMed. (DOCX 17 kb)

## Data Availability

The datasets used and/or analyzed during the current study are available from the corresponding author on reasonable request.

## Abbreviations

5-FU: 5-fluorouracil
CRC: colorectal cancer
Exo/P: exosomes from RKO/P
Exo/R: exosomes from RKO/R
HSFCM: high-sensitivity flow cytometer
RKO/P: RKO parental cell line
RKO/R: 5-FU resistant cell line
RTCA: Real-Time Cellular Analysis
TEM: transmission electron microscopy
WB: western blotting
