# Supplementary figures and images for: Exosomal transfer of p-STAT3 promotes acquired 5-FU resistance in colorectal cancer cells
Source: J Exp Clin Cancer Res. 2019 Jul 19;38:320. doi: 10.1186/s13046-019-1314-9 (PMC6642525; doi:10.1186/s13046-019-1314-9)

Figure S1

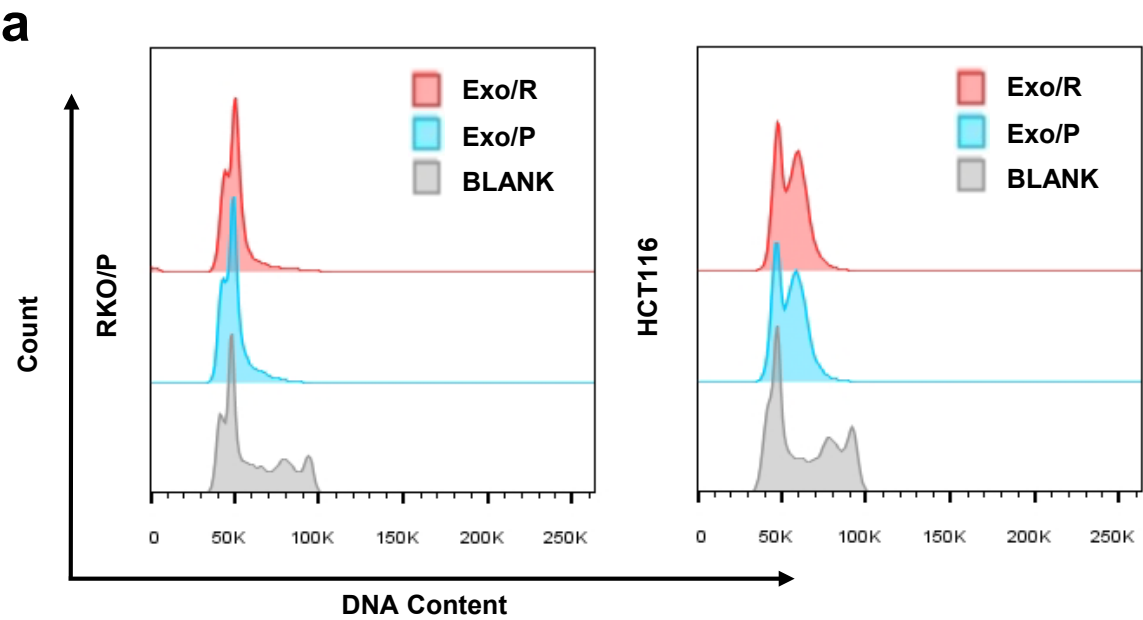

Figure S2

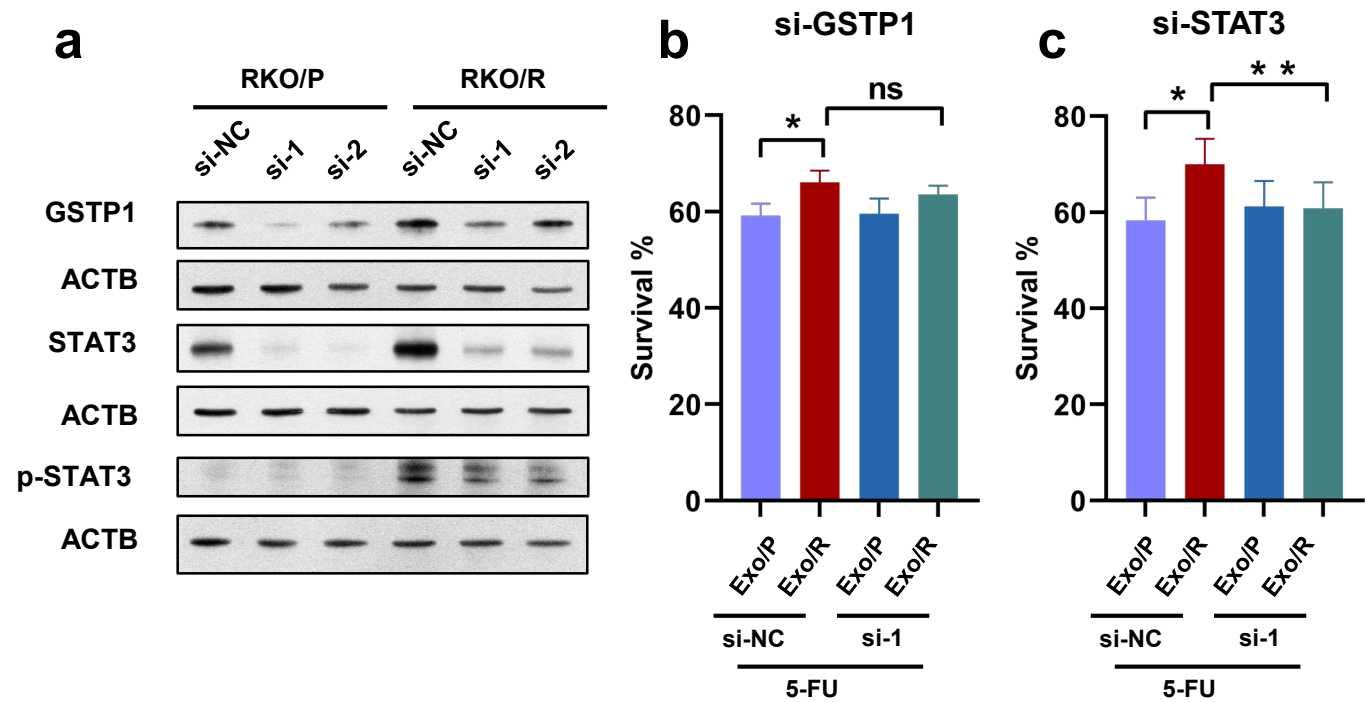

Supplement: Supplementary file 1 — Figure S1. The cell cycle distribution of RKO/P and HCT116 cells. a. Representative cell cycle changes of HCT116 and RKO/P cells exposed to 10 μM or 30 μM 5-FU and Exo/P or Exo/R after 24 hours. The DNA content was stained with PI. exposed to 10 μM or 30 μM 5-FU and Exo/P or Exo/R after 24 hours. The DNA content was stained with PI. Figure S2. p-STAT3 transferred by Exo/R p-STAT3 transferred by Exo/R mediated acquired 5-FU resistance in RKO/P cells. a. The WB result of GSTP1 and STAT3 in RKO/P and RKO/R cells transfected with si-NC, si-GSTP1 (si-1, si-2) and si-STAT3 (si-1, si-2). b. Statistical analysis of the survival rate of RKO/P cells treated with 30 μM 5-FU and exosomes from RKO/P or RKO/R cells after down-regulation of GSTP1. c. Statistical analysis of the survival rate of RKO/P cells treated with 30 μM 5-FU and exosomes from RKO/P or RKO/R cells after down-regulation of p-STAT3. *P < 0.05, **P < 0.01. (PDF 308 kb) [file 13046_2019_1314_MOESM1_ESM.pdf]
