# Supplementary material for: Exosomal transfer of p-STAT3 promotes acquired 5-FU resistance in colorectal cancer cells
Source: J Exp Clin Cancer Res. 2019 Jul 19;38:320. doi: 10.1186/s13046-019-1314-9 (PMC6642525; doi:10.1186/s13046-019-1314-9)
Supplement: Supplementary file 2 — Table S1. Overlapped parts in Venn diagram among RNA-seq, Proteomics and PubMed. (DOCX 17 kb) [file 13046_2019_1314_MOESM2_ESM.docx]

**Table S1** Overlapped parts in Venn diagram among RNA-seq, Proteomics and PubMed.

| 189 common elements in **RNA-seq** and **Proteomics** | 3 common elements in **RNA-seq** and **PubMed** | 2 common elements in **RNA-seq**, **Proteomics** and **PubMed** | 1 common element in **Proteomics** and **PubMed** |
| --- | --- | --- | --- |
| 43352 | MTHFR | STAT3 | ANXA1 |
| HIST1H2BN | BIRC3 | GSTP1 |  |
| HIST1H2BF | ABCB1 |  |  |
| RBM14-RBM4 |  |  |  |
| HBB |  |  |  |
| PGAM2 |  |  |  |
| GCA |  |  |  |
| KIF27 |  |  |  |
| RAB6B |  |  |  |
| ULBP1 |  |  |  |
| CROCC |  |  |  |
| NPIPB6 |  |  |  |
| HIST1H2BD |  |  |  |
| PKLR |  |  |  |
| HIST1H2BO |  |  |  |
| DPP8 |  |  |  |
| TMED7-TICAM2 |  |  |  |
| GNAL |  |  |  |
| NPIPB15 |  |  |  |
| HLA-G |  |  |  |
| GNAI1 |  |  |  |
| MFGE8 |  |  |  |
| CLU |  |  |  |
| TSPAN6 |  |  |  |
| ACTR3C |  |  |  |
| GSN |  |  |  |
| RCN1 |  |  |  |
| HIST1H2BJ |  |  |  |
| GAS6 |  |  |  |
| MYH14 |  |  |  |
| TWSG1 |  |  |  |
| NAV1 |  |  |  |
| LRRC1 |  |  |  |
| KRT15 |  |  |  |
| TES |  |  |  |
| TINAGL1 |  |  |  |
| ERO1B |  |  |  |
| CXCL3 |  |  |  |
| ATXN10 |  |  |  |
| PARVA |  |  |  |
| RAB5B |  |  |  |
| HLA-H |  |  |  |
| UGDH |  |  |  |
| LRRC16A |  |  |  |
| EGFR |  |  |  |
| ERBB2 |  |  |  |
| HIST1H2BC |  |  |  |
| CYB5B |  |  |  |
| PLAT |  |  |  |
| SLC2A1 |  |  |  |
| DNPEP |  |  |  |
| ACTA2 |  |  |  |
| TUBA4A |  |  |  |
| ATP1B1 |  |  |  |
| HMGB3 |  |  |  |
| ITGAV |  |  |  |
| PTBP3 |  |  |  |
| MBP |  |  |  |
| HSPH1 |  |  |  |
| LCN2 |  |  |  |
| JUP |  |  |  |
| IGFBP4 |  |  |  |
| DPYSL3 |  |  |  |
| BMP4 |  |  |  |
| ITGB4 |  |  |  |
| TACSTD2 |  |  |  |
| CXCL5 |  |  |  |
| EEF1A2 |  |  |  |
| KRT19 |  |  |  |
| NPNT |  |  |  |
| ST14 |  |  |  |
| FGFBP1 |  |  |  |
| DDX3Y |  |  |  |
| CLDN4 |  |  |  |
| RDX |  |  |  |
| CXCL1 |  |  |  |
| S100A14 |  |  |  |
| QPCT |  |  |  |
| TGFB2 |  |  |  |
| MATN2 |  |  |  |
| EIF3CL |  |  |  |
| PRSS8 |  |  |  |
| LGALS3BP |  |  |  |
| DKK1 |  |  |  |
| CDH1 |  |  |  |
| CEMIP |  |  |  |
| NEBL |  |  |  |
| EPCAM |  |  |  |
| CD14 |  |  |  |
| KRT8 |  |  |  |
| THBS1 |  |  |  |
| EPN3 |  |  |  |
| EPS8L2 |  |  |  |
| CXCL2 |  |  |  |
| KLK10 |  |  |  |
| TIMP2 |  |  |  |
| PCSK9 |  |  |  |
| SMPDL3B |  |  |  |
| CILP2 |  |  |  |
| FBXO2 |  |  |  |
| ANXA3 |  |  |  |
| DSP |  |  |  |
| NOTUM |  |  |  |
| FAT1 |  |  |  |
| S100A10 |  |  |  |
| HLA-B |  |  |  |
| MYH10 |  |  |  |
| TSPAN5 |  |  |  |
| KRT18 |  |  |  |
| SERINC5 |  |  |  |
| LAMC2 |  |  |  |
| SLC12A2 |  |  |  |
| MYOF |  |  |  |
| LGALS3 |  |  |  |
| MARVELD3 |  |  |  |
| MBD5 |  |  |  |
| SERPINB9 |  |  |  |
| NQO1 |  |  |  |
| SLC44A2 |  |  |  |
| PLAU |  |  |  |
| HIST1H2BK |  |  |  |
| TUBB3 |  |  |  |
| TUBB6 |  |  |  |
| AGRN |  |  |  |
| F3 |  |  |  |
| LLGL2 |  |  |  |
| MYO6 |  |  |  |
| ITGA6 |  |  |  |
| ITGA3 |  |  |  |
| CD82 |  |  |  |
| F11R |  |  |  |
| FGFRL1 |  |  |  |
| PLXNB2 |  |  |  |
| LSR |  |  |  |
| CKAP4 |  |  |  |
| APRT |  |  |  |
| CUL4B |  |  |  |
| DPYSL2 |  |  |  |
| VCL |  |  |  |
| SFN |  |  |  |
| LMAN1 |  |  |  |
| UBA2 |  |  |  |
| DNAJB11 |  |  |  |
| LMNA |  |  |  |
| EIF4A2 |  |  |  |
| PTK7 |  |  |  |
| PGM1 |  |  |  |
| DLG1 |  |  |  |
| MYO1B |  |  |  |
| DAG1 |  |  |  |
| RCC2 |  |  |  |
| PPCS |  |  |  |
| SPINT1 |  |  |  |
| NUMA1 |  |  |  |
| ANXA11 |  |  |  |
| VPS35 |  |  |  |
| ACTN4 |  |  |  |
| ICAM1 |  |  |  |
| ATP6V1A |  |  |  |
| NECTIN2 |  |  |  |
| FDPS |  |  |  |
| SYNCRIP |  |  |  |
| HIST1H1C |  |  |  |
| CNN2 |  |  |  |
| GRHPR |  |  |  |
| IK |  |  |  |
| HLA-A |  |  |  |
| RNPEP |  |  |  |
| GANAB |  |  |  |
| ETFB |  |  |  |
| HSP90B1 |  |  |  |
| EIF3J |  |  |  |
| RACGAP1 |  |  |  |
| KTN1 |  |  |  |
| BZW1 |  |  |  |
| NDUFB10 |  |  |  |
| QSOX1 |  |  |  |
| CTNND1 |  |  |  |
| LMNB1 |  |  |  |
| G6PD |  |  |  |
| EIF3C |  |  |  |
| RPN1 |  |  |  |
| PDIA4 |  |  |  |
| CD9 |  |  |  |
| PTMA |  |  |  |
| EIF4G2 |  |  |  |
| TUBA1B |  |  |  |
| ATP1A1 |  |  |  |
| PCBP2 |  |  |  |
